# Re-analysis of long non-coding RNAs and prediction of circRNAs reveal their novel roles in susceptible tomato following TYLCV infection

**DOI:** 10.1186/s12870-018-1332-3

**Published:** 2018-06-04

**Authors:** Jinyan Wang, Yuwen Yang, Lamei Jin, Xitie Ling, Tingli Liu, Tianzi Chen, Yinghua Ji, Wengui Yu, Baolong Zhang

**Affiliations:** 1 Provincial Key Laboratory of Agrobiology, Institute of Crop Germplasm and Biotechnology, Jiangsu Academy of Agricultural Sciences, Nanjing, 210014 Jiangsu China; 2 Institute of Plant Protection, Jiangsu Academy of Agricultural Sciences, Nanjing, 210014 Jiangsu China

**Keywords:** Susceptible tomato, Long non-coding RNA, circRNA, TYLCV

## Abstract

**Background:**

Long Noncoding-RNAs (LncRNAs) are known to be involved in some biological processes, but their roles in plant-virus interactions remain largely unexplored. While circular RNAs (circRNAs) have been studied in animals, there has yet to be extensive research on them in a plant system, especially in tomato-tomato yellow leaf curl virus (TYLCV) interaction.

**Results:**

In this study, RNA transcripts from the susceptible tomato line JS-CT-9210 either infected with TYLCV or untreated, were sequenced in a pair-end strand-specific manner using ribo-zero rRNA removal library method. A total of 2056 lncRNAs including 1767 long intergenic non-coding RNA (lincRNAs) and 289 long non-coding natural antisense transcripts (lncNATs) were obtained. The expression patterns in lncRNAs were similar in susceptible tomato plants between control check (CK) and TYLCV infected samples. Our analysis suggested that lncRNAs likely played a role in a variety of functions, including plant hormone signaling, protein processing in the endoplasmic reticulum, RNA transport, ribosome function, photosynthesis, glulathione metabolism, and plant-pathogen interactions. Using virus-induced gene silencing (VIGS) analysis, we found that reduced expression of the lncRNA S-slylnc0957 resulted in enhanced resistance to TYLCV in susceptible tomato plants. Moreover, we identified 184 circRNAs candidates using the CircRNA Identifier (CIRI) software, of which 32 circRNAs were specifically expressed in untreated samples and 83 circRNAs in TYLCV samples. Approximately 62% of these circRNAs were derived from exons. We validated the circRNAs by both PCR and Sanger sequencing using divergent primers, and found that most of circRNAs were derived from the exons of protein coding genes. The silencing of these circRNAs parent genes resulted in decreased TYLCV virus accumulation.

**Conclusion:**

In this study, we identified novel lncRNAs and circRNAs using bioinformatic approaches and showed that these RNAs function as negative regulators of TYLCV infection. Moreover, the expression patterns of lncRNAs in susceptible tomato plants were different from that of resistant tomato plants, while exonic circRNAs expression positively associated with their respective protein coding genes. This work provides a foundation for elaborating the novel roles of lncRNAs and circRNAs in susceptible tomatoes following TYLCV infection.

**Electronic supplementary material:**

The online version of this article (10.1186/s12870-018-1332-3) contains supplementary material, which is available to authorized users.

## Background

With developments in genomics, especially in applications of next-generation high-throughput sequencing technology, there has been an unprecedented increase in the ability to detect unknown transcripts. These transcripts, which appear to not be derived from known annotated protein-coding genes, are identified as non-coding RNAs (ncRNAs). A wide variety of non-coding RNA exist in plant cells including small interfering RNA (siRNA), microRNA (miRNA), *trans*-acting siRNA (tasiRNA), long non-coding RNA (lncRNA), and a diverse and abundant class of endogenous non-canonical RNAs called circular RNA (circRNA) [1–5]. LncRNAs are typically longer than 200 nucleotides and primarily transcribed by RNA polymerase II (Pol II) or III. Most lncRNAs are capped, polyadenylated or non-polyadenylated, and spliced or non-spliced [6]. With the rapid development of next-generation sequencing technology, a number of lncRNAs have recently been identified in a variety of plant species via bioinformatic analysis, including Arabidopsis [7–10], rice [11], wheat [12], tomato [13–15], cotton [16], cucumber [17], maize, Chinese cabbage [18], and Poplar [19, 20]. However, the functions of lncRNAs have not been well studied. Regulation of vernalization in Arabidopsis by two non-coding RNA, cool-assisted intronic non-coding RNA (COOLAIR) and cold-assisted intronic non-coding RNA (COLDAIR), transcribed by Flowering Locus C (FLC), have already been elucidated [21–23]. The Arabidopsis non-coding RNA, HID1 (HIDDEN TREASURE 1) promotes photomorphogenesis and plays an important role in continuous red light [24]. A rice long non-coding RNA LDMAR is required for normal pollen development of rice grown under long-day conditions in photoperiod-sensitive male sterility lines [25]. In a photoperiod-sensitive male sterility line, there was a locus which also encoded long non-coding RNA PMS1T which was targeted by miR2118 to produce 21-nt phasiRNAs [26].

Circular RNAs (circRNAs) are a type of RNA produced from precursor mRNAs (pre-mRNAs) through backsplicing, a process in which a 3′ splicing acceptor site is joined to 5′ splicing donor site to, form a covalently closed loop [27]. There are also reports of circRNAs being present in intron and intergenic regions [28, 29]. Competition between canonical splicing and backsplicing within cells leads to a lower abundance of circRNAs as compared to the corresponding linear mRNAs [30]. Recent studies have demonstrated that alternative splicing (AS) events are involved in the biosynthesis of circRNAs [31]. Recently several bioinformatics pipelines have been developed to predict circRNAs based on next-generation sequencing datasets, such as circRNA_finder [32], find_circ [5], CIRIexplorer [33], CIRI [34], and MapSplice [35]. Using these algorithms, circRNAs abundance has been observed in plants, such as in Arabidopsis [36], rice [37], wheat [38], kiwifruit [39], and tomato [40]. However, the functions of circRNAs in plants are still largely unknown. Recent findings in animals studies have suggested that circRNAs act as competing endogenous RNA, or miRNA sponges that protect miRNA targets [41]. Therefore, interactions between circRNA, miRNA, and mRNA are considered as the primary expression pattern regulators in transcriptional and post-transcriptional regulation. In addition, circRNAs may also affect AS, which leads to altered parent gene expression because their formation positively correlates with exons skipped in linear mRNAs [30].

Tomato (*Solanum lycopersicum*) is among the most widely grown and economically important vegetable crops in the world [42]. *Tomato yellow leaf curl virus* (TYLCV) is a begomovirus (genus *Begomovirus*, family *Geminiviridae*) that is transmitted by the whitefly *Bemisia tabaci*. It is one of the most damaging viruses (rank 3rd in the world) for tomato plants [43]. Multiple loci that are tightly linked to TYLCV resistance (*Ty-1* to *Ty-6*) were mapped onto tomato chromosomes by molecular markers. Of these, *Ty-1*, *Ty-3*, and *Ty-5* had been cloned in different wild tomato accessions, and *Ty-1* and *Ty-3* were found to be allelic. They were identified and shown as RNA-dependent RNA polymerases (RDRs) that may be involved in virus RNA silencing [44]. Pelota (Pelo) encodes the tomato homolog of the messenger mRNA surveillance factor and controls TYLCV resistance as the *Ty-5* locus [45]. Meanwhile, a few tomato transcription factors have been shown to respond to TYLCV infection, such as basic helix-loop-helix (bHLH) [46], AP2/ERF [47], and WRKY Group III [48].

In a previous study, 1565 lncRNAs were identified in the TYLCV-resistant tomato breeding line CLN2777A and several lncRNAs acted as competing endogenous target mimics (eTMs) for tomato microRNA response to TYLCV infection [13]. In addition, 854 circRNAs were identified using deep sequencing and bioinformatics, of which 163 circRNAs exhibited chilling responsive expression. Among them, 102 circRNAs were found to be sponges for 24 miRNAs [40]. However, there is no evidence which shows the role of circRNAs in tomato plant TYLCV infection. Moreover, the role of lncRNAs in TYLCV-susceptible tomato plants is still unknown. To explore lncRNAs and circRNAs in TYLCV-susceptible tomato and their potential function in the regulation of the TYLCV response, we identified lncRNAs and circRNAs at a genome wide scale in TYLCV-tomato leaves using strand-specific RNA sequencing technology [14]. Our results suggested that several lncRNAs act as a susceptibility gene in TYLCV infection, and some exonic circRNAs positively associate with the expression of parent genes.

## Results

### Identification of lncRNAs in susceptible tomatoes with TYLCV infection

**Fig. 1:**
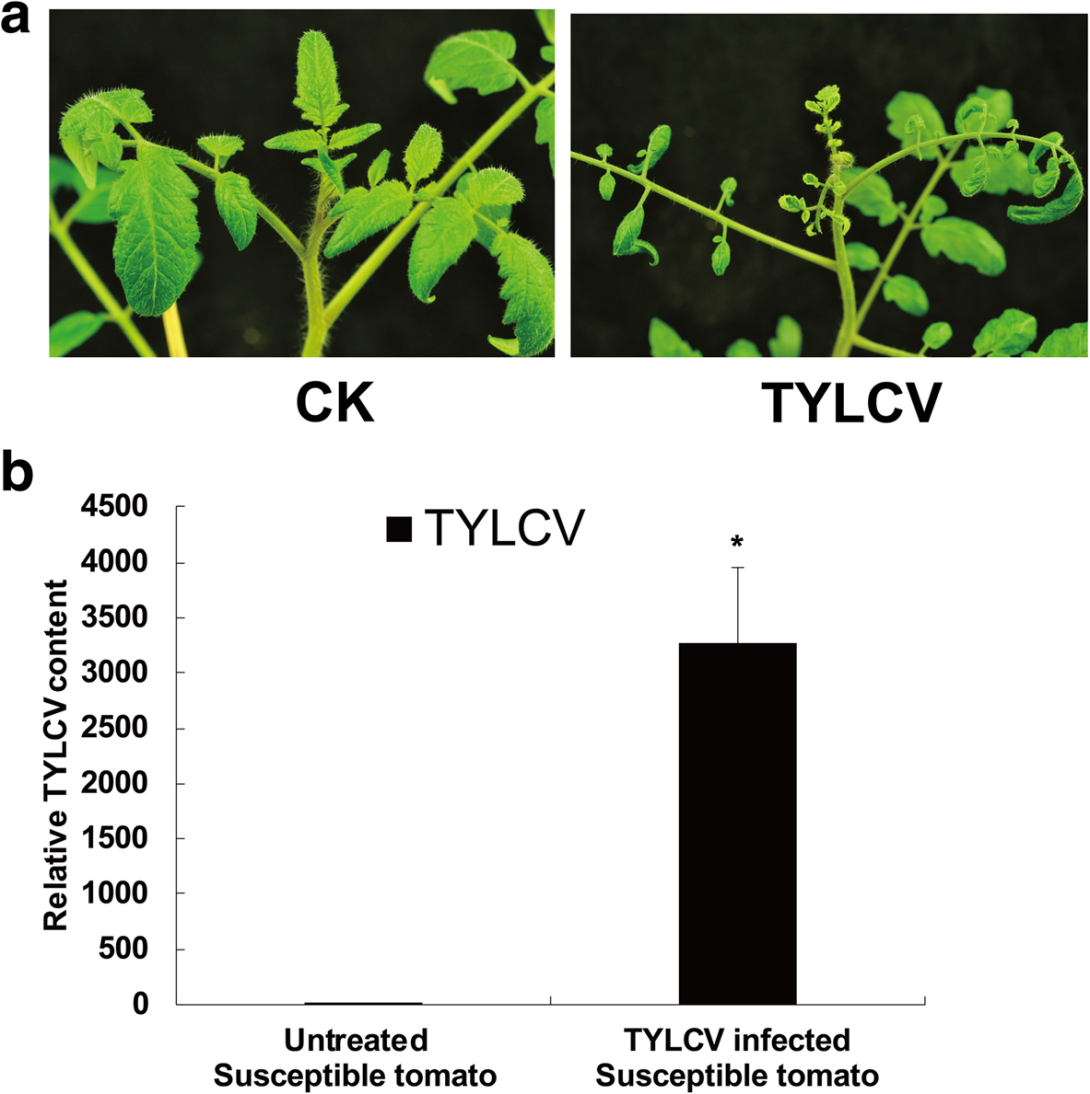
Leaf phenotype and TYLCV DNA accumulation 21 days after TYLCV infection of the susceptible tomato lines JS-CT-9210. **a** The new merging leaves phenotype. CK is untreated tomato plants. TYLCV is tomato plants with TYLCV infection. **b** The TYLCV DNA accumulation in new emerging leaves measured by qPCR. Tomato α-tubulin (Solyc04g077020.2) was used as an internal reference. Error bars represented standard errors of three biological replicates, and asterisks indicate significant differences based on the Student’s t test (*P* < 0.05)

The susceptible tomato line JS-CT-9210 showing obvious symptoms of TYLCV infection was used for library preparation and RNA sequencing. At 21 dpi, new emerging leaves were curly, mottled and yellow (Fig. 1a), and TYLCV accumulation in TYLCV susceptible plants was extremely high (Fig. 1b). Using poly (A) enrichment RNA sequencing, a previous study predicted 1565 lncRNAs including lincRNAs and lncNATs in TYLCV infected resistant tomato plants [13]. However, in that study some lncRNAs without a poly (A) tail were not identified due to the RNA enrichment method in library preparation step. To identify novel lncRNAs, pair-end strand-specific RNA sequencing was performed using the ribo-zero rRNA removal library method on three biological replicates from samples off the susceptible tomato line JS-CT-9210, either inoculated with TYLCV or untreated. A total of ~ 395 million reads were obtained (Table 1), and the base quality of reads was checked using FastQC (Additional file 1: Figure S1). Based on the value of fragments per kilobase of exon per million fragments mapped (FPKM), we calculated the coefficient of correlation in three repeats for each sample. The results indicated that there was a perfect correlation among the three biological replicates, and the Pearson’s correlation for almost all comparisons was larger than 97% (Additional file 2: Figure S2).

**Table 1:** The summary of RNA-seq reads

	Clean data	Mapped Unique Left Reads	Mapped Unique Right Reads	Total mapping pair reads	Overall mapping
CK-1	66,880,823	60,045,974	55,871,998	54,219,416	81.00%
CK-2	73,056,020	65,657,353	62,301,119	60,463,488	82.70%
CK-3	60,689,421	53,980,599	50,186,626	48,688,695	80.20%
TYLCV-1	69,064,126	61,832,635	57,941,991	56,295,568	81.50%
TYLCV-2	62,050,789	54,935,875	51,168,442	49,630,915	79.90%
TYLCV-3	64,256,653	57,864,898	54,713,490	53,138,232	82.60%

**Fig. 2:**
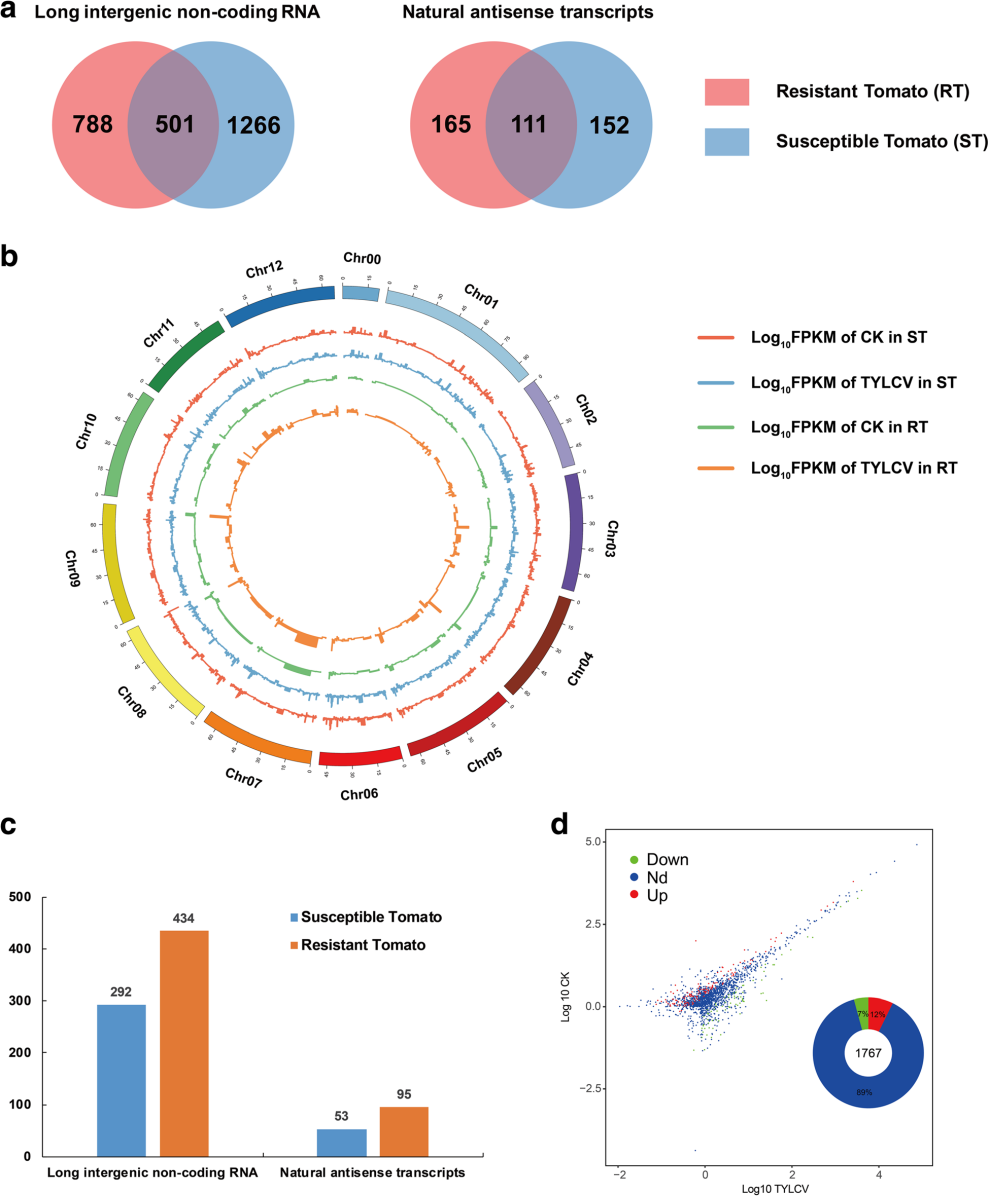
Characterization and comparison of lncRNAs in susceptible tomato (ST) and resistant tomato plants (RT). **a** A Venn diagram showing the number of lncRNAs between RT and ST. **b** The expression level of lncRNAs (Log10 FPKM) along 12 tomato chromosomes in RT and ST. **c** The number of differentially expressed lncRNAs in ST and RT. **d** Scatter plot of comparative results of log transformed gene differentially gene expression level between CK and TYLCV. The red dot represents up-regulated gene; the green dot represents down-regulated gene; the blue dot represents the non-differentially expressed gene

To identify potential novel lncRNAs, certain sequential stringent filters were used as described in a previous study [13]. However, few filter criteria were modified in the present study due to the larger quantity of sequencing data. The expression level with FPKM value in lncRNA candidates were higher than 2 for single-exon transcript or 1 for multiple-exon transcripts. Next, lncRNA candidates were assessed for protein-coding potential by the Coding Potential Calculator (CPC) and Coding-Non-Coding Index (CNCI) programs. Finally, a total of 2056 lncRNAs including 1767 lincRNAs (Additional file 3: Table S1) and 289 lncNATs (Additional file 4: Table S2) were obtained. In comparison with a previous study, 501 lincRNAs and 111 lncNATs were identical to resistant tomatoes. Furthermore, 1266 lincRNA and 152 lncNATs were specific in susceptible tomatoes (Fig. 2a). Next the lncRNAs were mapped on to the tomato genome SL2.50. A circos plot showed that these lncRNAs were more evenly distributed across whole 12 chromosomes (Fig. 2b). The expression patterns (Log10 FPKM) in lncRNAs were similar in susceptible tomatoes between CK and TYLCV samples, and this trend was also observed in resistant tomatoes. To identify additional TYLCV-related lncRNAs, the expression levels were compared and a total of 345 lncRNAs were significantly differentially expressed between CK and TYLCV samples (Fig. 2c). Also, the number of differentially expressed lncRNAs (DELs) in susceptible tomatoes was lower than in resistant tomatoes. Among these DELs, 212 lncRNAs (7%) were up-regulated and 124 lncRNAs (12%) were down-regulated (Fig. 2d).

### Functional analysis of lncRNAs in susceptible tomatoes

**Fig. 3:**
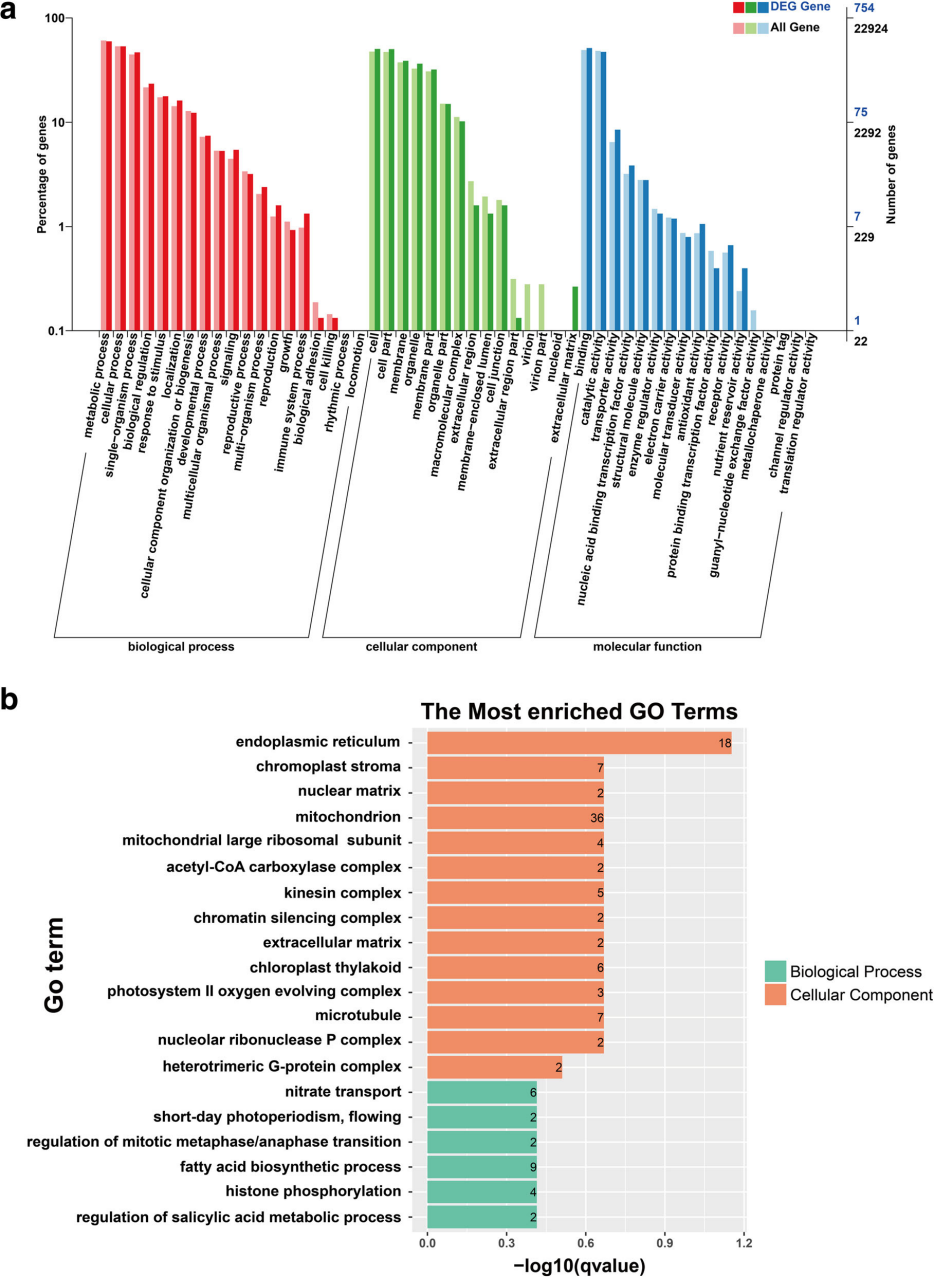
Gene ontology (GO) for neighboring genes of lncRNAs (**a**) Gene categories disctribution of lncRNAs under molecular functions, cellular components, and biological processes. **b** The most enriched GO terms of lncRNAs in biological process and cellular component

A previous study showed that lncRNAs may preferentially regulate neighboring genes [15]. To reveal potential functions of lncRNA candidates, we identified their co-localization genes and DEGs within the neighboring 100 kb region and analyzed GO terms and KEGG pathway enrichment of these genes. As shown in Fig. 3a, a total of 22,924 neighboring genes and 754 DEGs were assigned at least one GO term. The most highly represented categories within GO molecular function were ‘binding’ (GO: 0005488) and ‘catalytic activity’ (GO: 0005488). For GO cellular component, genes involved in ‘cell’ (GO: 0005623) and ‘cell part’ (GO: 0044464) were the most highly represented. For the biological processes, the two highest represented categories were ‘metabolic process’ (GO: 0008152) and ‘cellular process’ (GO: 0009987). To better understand enriched GO terms for neighboring genes of lncRNAs, we further investigated the biological processes and cellular component classifications and found that endoplasmic reticulum (ER) was over-represented under cellular component. Furthermore, biological processes such as salicylic acid metabolic process, fatty acid biosynthetic process and nitrate transport were the most highly represented (Fig. 3b).

**Fig. 4:**
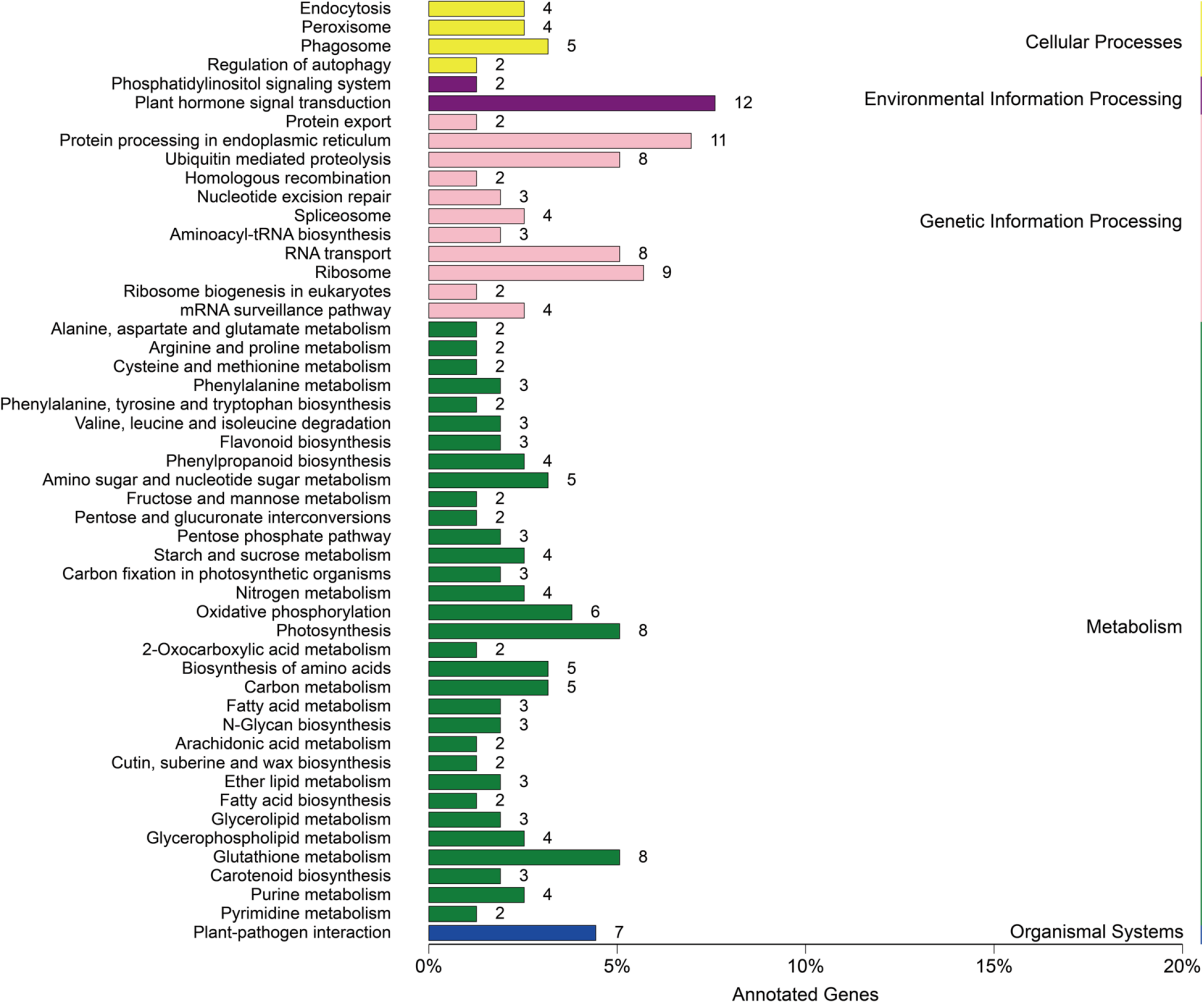
KEGG pathways enrichment of neighboring genes of lncRNAs in cellular processes, environmental information processing, genetic information processing, metabolism and organismal systems

In addition, we mapped neighboring genes of lncRNAs to the KEGG database to identify significantly enriched pathways. In these mapped pathways, 13 pathways were enriched with more than five genes. These pathways were distributed in five major categories, including cellular processes, environmental information processing, genetic information processing, metabolism, and organismal systems. Notably, common enrichments were observed in plant hormone signaling system, protein processing in endoplasmic reticulum, RNA transport, ribosome, photosynthesis, glutathione metabolism, and plant-pathogen interaction (Fig. 4). These findings indicated that TYLCV infection in tomatoes affected the expression of genes involved in these pathways. We also analyzed the DEGs in neighboring genes that belonged to KEGG enrichment categories. The results showed that 20 pathways were enriched, especially those involved in nitrogen metabolism (Additional file 5: Figure S3).

### Differential expression of lncRNAs in susceptible tomato

**Fig. 5:**
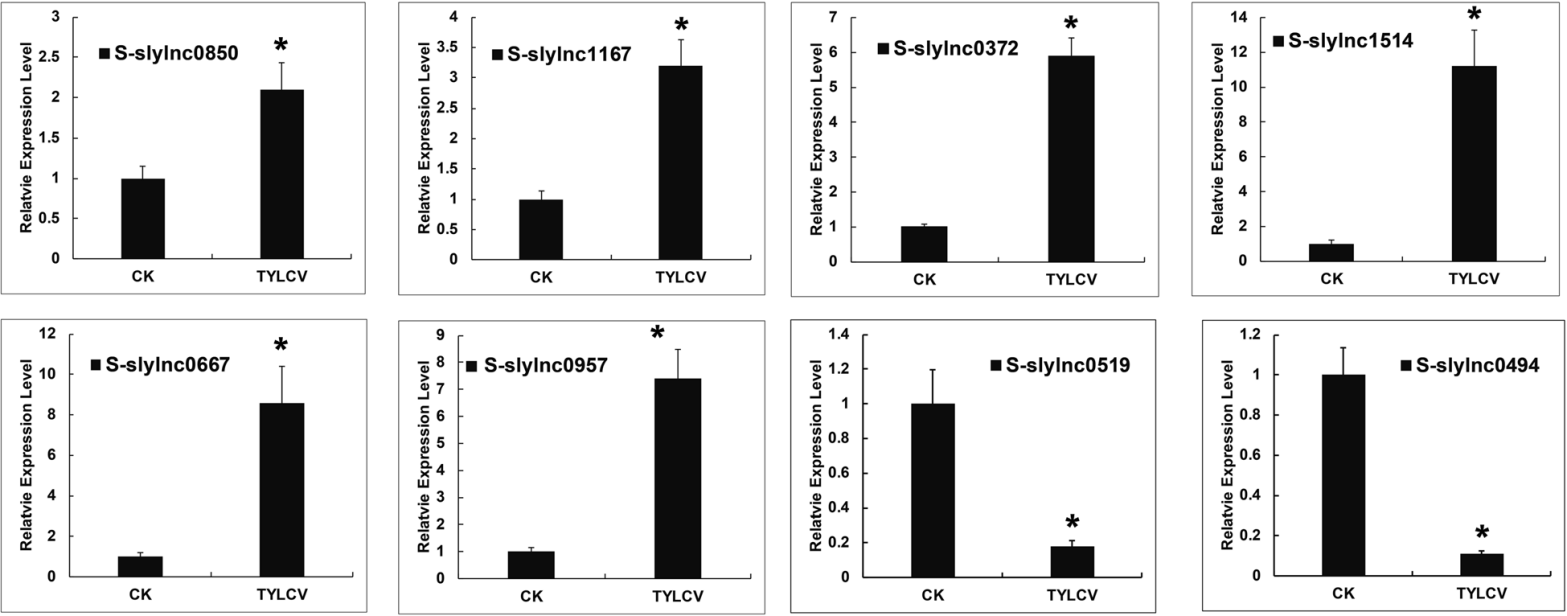
Confirmation of the expression patterns of differentially expressed lncRNAs using quantitative RT-PCR. Tomato α-tubulin (Solyc04g077020.2) was used as an internal reference. Error bars represented the standard error of three biological replicates. Asterisks indicate significant differences by Student’s t test (*P* < 0.05)

To validate the expression of lncRNAs in susceptible tomato plants after TYLCV infection, we selected eight lncRNAs (S-slylnc0850, S-slylnc1167, S-slylnc0372, S-slylnc1514, S-slylnc0667, S-slylnc0957, S-slylnc0519 and S-slylnc0494) that were only expressed in susceptible tomatoes with greater than 2-fold change and *p*-values < 0.05 as experimental validation and determined their expressions by qRT-PCR. The RNA-seq expression patterns were consistent with the qRT-PCR results as seen in six lncRNAs with up-regulated expression and two lncRNAs with down-regulated expression (Fig. 5). Furthermore, fold change between RNA-seq and qRT-PCR data were positively correlated (R^2^ = 0.94, *P* value < 0.05) (Additional file 6: Figure S4). These results indicated that our RNA sequencing data was reliable and that the identified lncRNAs were indeed TYLCV-related lncRNAs, further suggesting that these lncRNA may play important roles in response to TYLCV infection.

### Gene silencing of lncRNA can promote tomato resistance to TYLCV infection

**Fig. 6:**
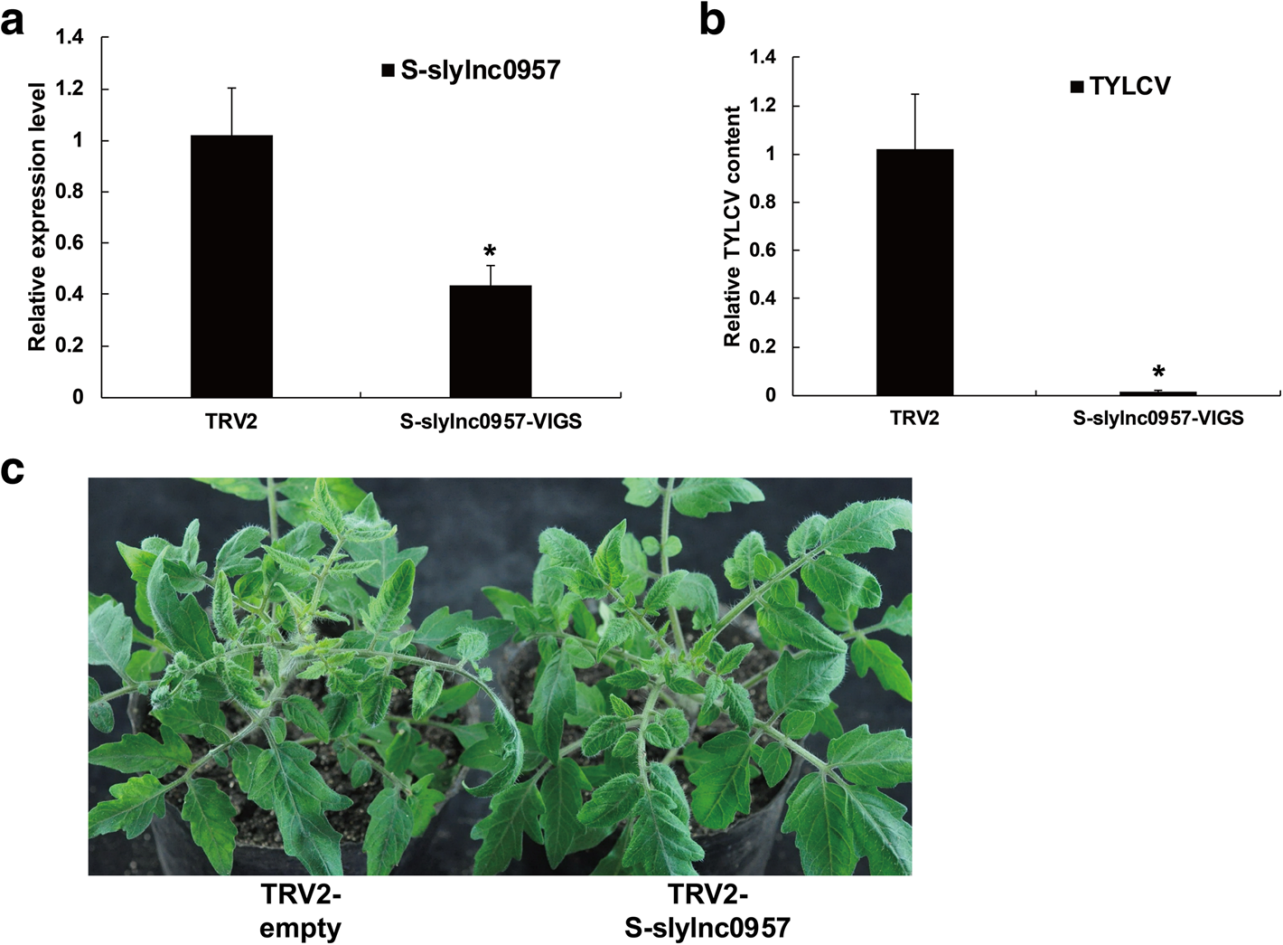
Validation of lncRNAs with virus-induced gene silencing. **a** Relative expression levels of S-slylnc0957 using real-time RT-PCR analysis in the VIGS-treated tomato plants 20 days after agroinfiltration with TRV2 vectors. Tomato α-tubulin (Solyc04g077020.2) was used as an internal reference. Error bars represented standard errors of three biological replicates, and asterisks indicate significant differences based on the Student’s t test (*P* < 0.05). **b** TYLCV accumulation in the S-slylnc0957 silenced plants was estimated from the total genomic DNA by quantitative RT-PCR. Values were normalized using tomato α-tubulin (Solyc04g077020.2) as an internal reference. Error bars represented standard errors of three biological replicates and asterisk indicates significant difference based on the Student’s t test (*P* < 0.05). **c** Cotyledon agroinfiltration of TRV vectors with TYLCV infection was performed in the susceptible tomato at the cotyledon stage. Plants treated with the pTRV vectors showed the susceptible phenotype (Left). Susceptible plantlets treated with the S-slylnc0957 gene silencing constructs pTRV1 and pTRV2-S-slylnc0957 have no symptoms (right)

We investigated a possible role for differential expressed lncRNAs in resistance to TYLCV using VIGS in susceptible tomato plants. A TRV vector carrying a fragment of S-slylnc0957 was injected into tomato leaves at the cotyledon stage. These tomato plants were infected with the TYLCV infectious clone 14 days after VIGS. One month after VIGS, silencing was validated by appearance of pTRV-PDS. These results indicated that the VIGS system can be used to reduce the expression of lncRNAs. Quantitative RT-PCR was used to validate the effect of S-slylnc0957 silencing, and the results showed that the expression level of S-slylnc0957 decreased by more than 60% compared with the negative control (Fig. 6a). Also, virus accumulation of TYLCV-infected tomatoes was reduced more than 90% in the VIGS-treated tomato plants compared with the negative control (Fig. 6b), and symptoms of VIGS-treated tomato plants lessened in contrast to the negative control with empty TRV-vector in susceptible tomato plants (Fig. 6c). These results suggested that the lncRNA S-slylnc0957 acted as a negative regulator in response to TYLCV infection, and the silencing of this lncRNA can increase resistance to TYLCV in susceptible tomato plants.

### Identification of tomato circular RNAs

**Fig. 7:**
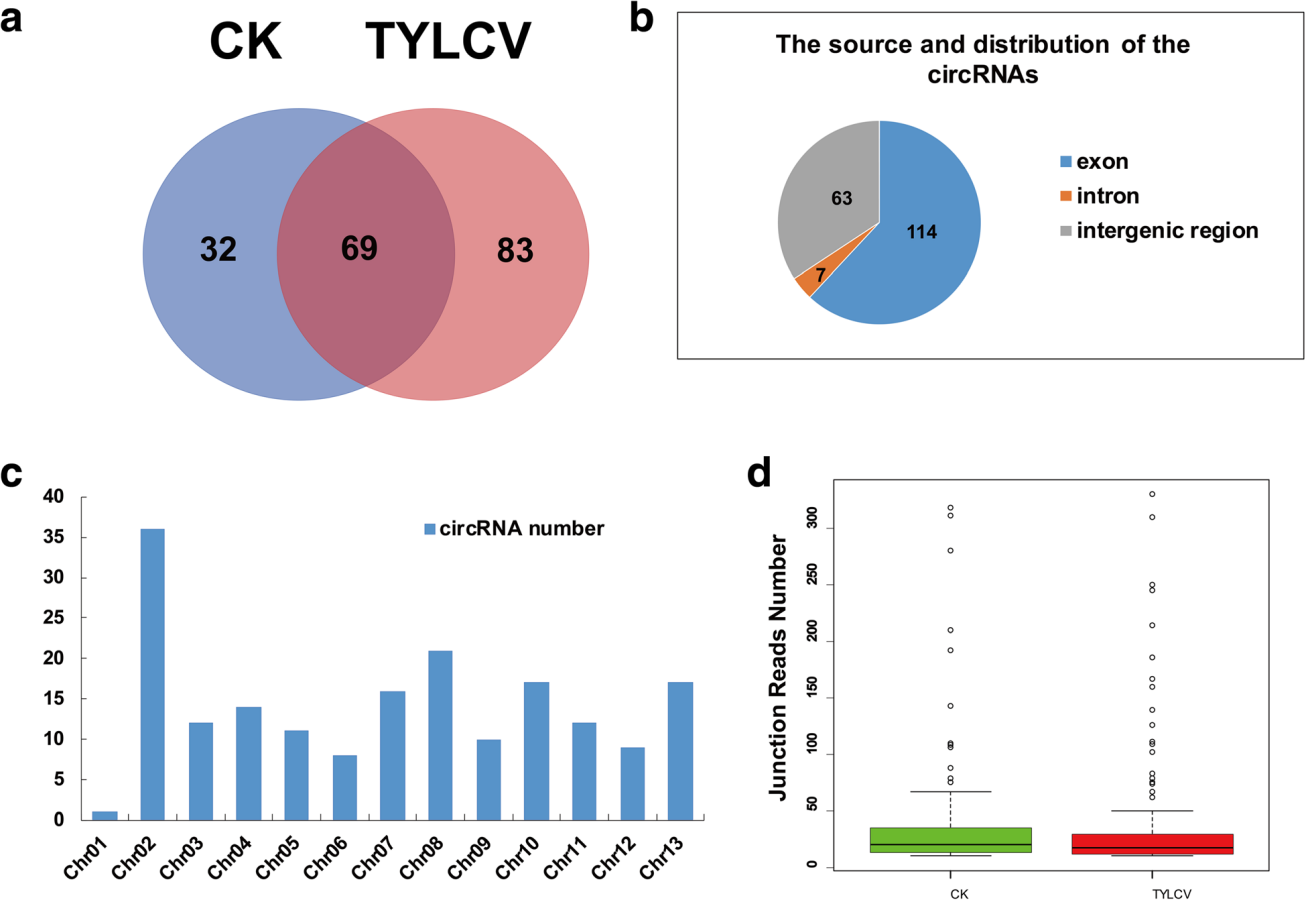
Characterization of circRNAs in CK and TYLCV samples. **a** The number of circRNAs in CK and TYLCV. **b** The circRNAs type according to their genomic location. **c** Distribution of circRNAs number in 12 tomato chromosomes. **d** The box plot of back-splice junction reads number between CK and TYLCV samples

In a previous study, the identification of circRNAs required more sequencing depth, preferably hundreds of millions reads [37]. To identify more circular RNA (circRNA) in TYLCV infected tomatoes, we assembled three biological replicates into one larger data set in CK and TYLCV samples, respectively. A total of more than 200 and 195 million pair-end reads in two samples were used to predict circRNA using the BWA-MEM software with the default parameter. Next, the resulting SAM file for alignment was used to recognize back-spliced junctions (BSJs) and CIRI (v2.0.2) was used to predict circRNA candidates. We also manually retained circRNAs with relatively high expression levels (read number of BSJs ˃ 10). Finally, 184 circRNA candidates were identified by the CIRI in the two samples of CK and TYLCV. We found 32 and 83 circRNAs that were specifically expressed in the CK and TYLCV samples, respectively (Fig. 7a, Additional file 7: Table S3). These circRNAs were divided into three groups, exonic circRNAs, intronic circRNAs, and intergenic circRNAs, on the basis of their genomic region origin (Fig. 7b). Among them, 114 (62%) circRNAs were derived from exons that were generated from a single protein-coding gene, and 63 (34.2%) circRNAs were generated from intergenic regions (intergenic circRNAs). Additionally, seven (3.8%) circRNAs were derived from introns (intronic circRNAs) (Fig. 7b). These circRNAs were mapped onto the 12 tomato chromosomes with an obviously uneven distribution, and chromosome 01 had the most circRNAs, whereas chromosome 05 had the least circRNAs (Fig. 7c). In terms of the junction reads number, circRNA in CK samples ranged from 10 to 311 with an average number of 45, while on average 35 reads were associated with circRNA in TYLCV sample, indicating that the expression of circRNAs in CK samples were higher than in TYLCV samples (Fig. 7d).

### Validation of tomato circRNAs

**Fig. 8:**
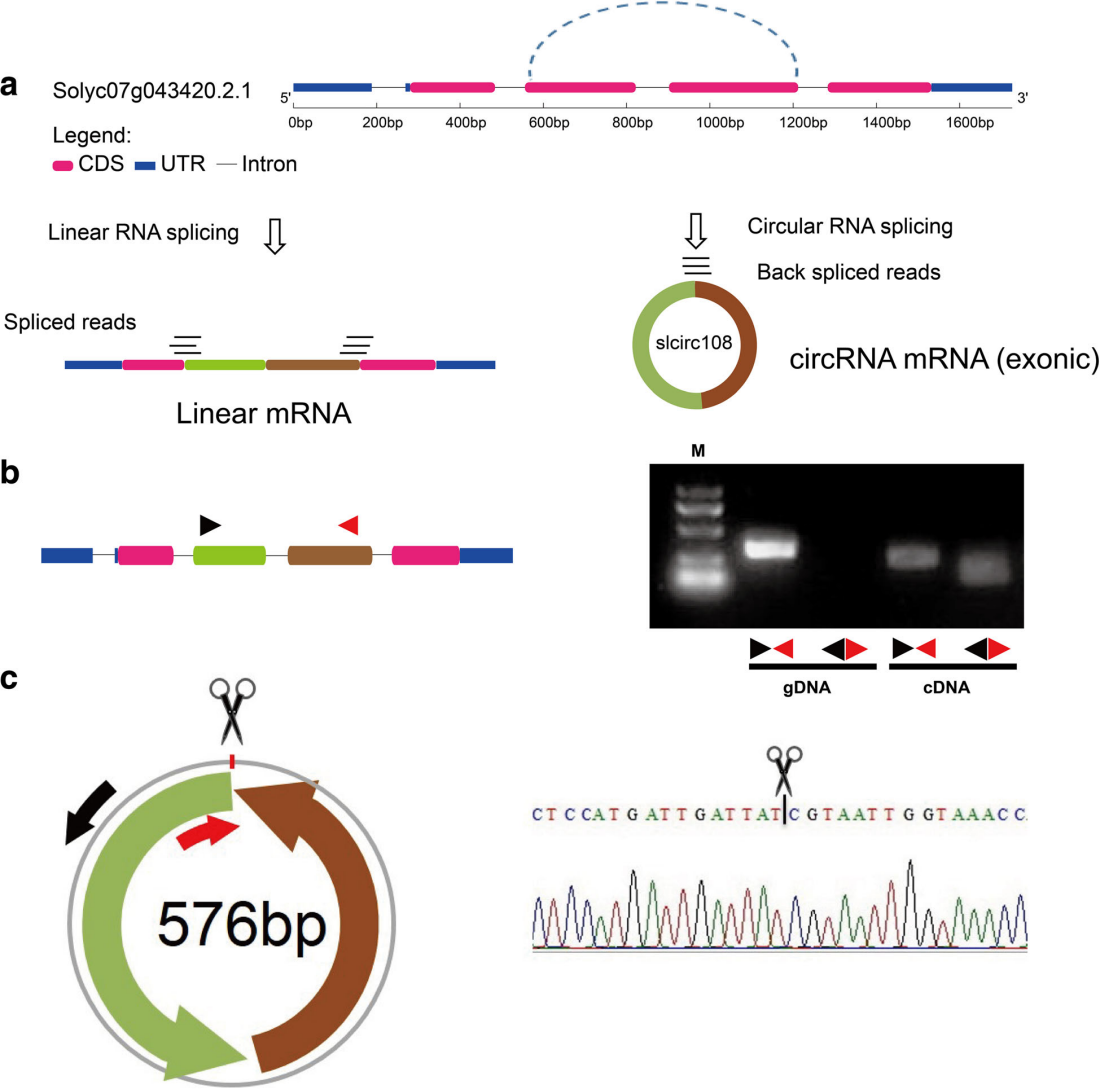
Characterization and validation of slcirc108. **a** The gene structure of slcirc108 parent gene Solyc07g043420.2.1, and aligned reads in linear RNA and circRNA. **b** The PCR amplification by divergent and convergent primers in genomic DNA and cDNA samples of tomato. **c** The Sanger sequencing of slcirc108 in back-splice junction

As described earlier, we identified 184 novel circRNAs in ribosome-depleted samples from CK and TYLCV infected leaves. Of them, we randomly selected circRNAs and experimentally tested the predictions including their expressions and back splicing sites using PCR and Sanger sequencing. Divergent primers and convergent primers were designed to amplify seven circRNAs in total RNA and genomic DNA (Additional file 8: Table S4). Additionally, slcirc108 was derived from the second and third exon of its parent protein-coding gene Solyc07g043420.2.1, and 186 junction-spanning reads and the backsplice junction were detected in TYLCV samples (Fig. 8a). Divergent primers can amplify circRNA in cDNA but not in DNA by PCR, while convergent primers can amplify products both in cDNA and DNA (Fig. 8b). Additionally, PCR amplification products from divergent primers by Sanger sequencing validated the predicted backsplice site (Fig. 8c). An additional six circRNAs were also validated by amplification of divergent primers and Sanger sequencing (Additional file 9: Figure S5).

### Correlation of expression between circRNAs and parent genes

**Fig. 9:**
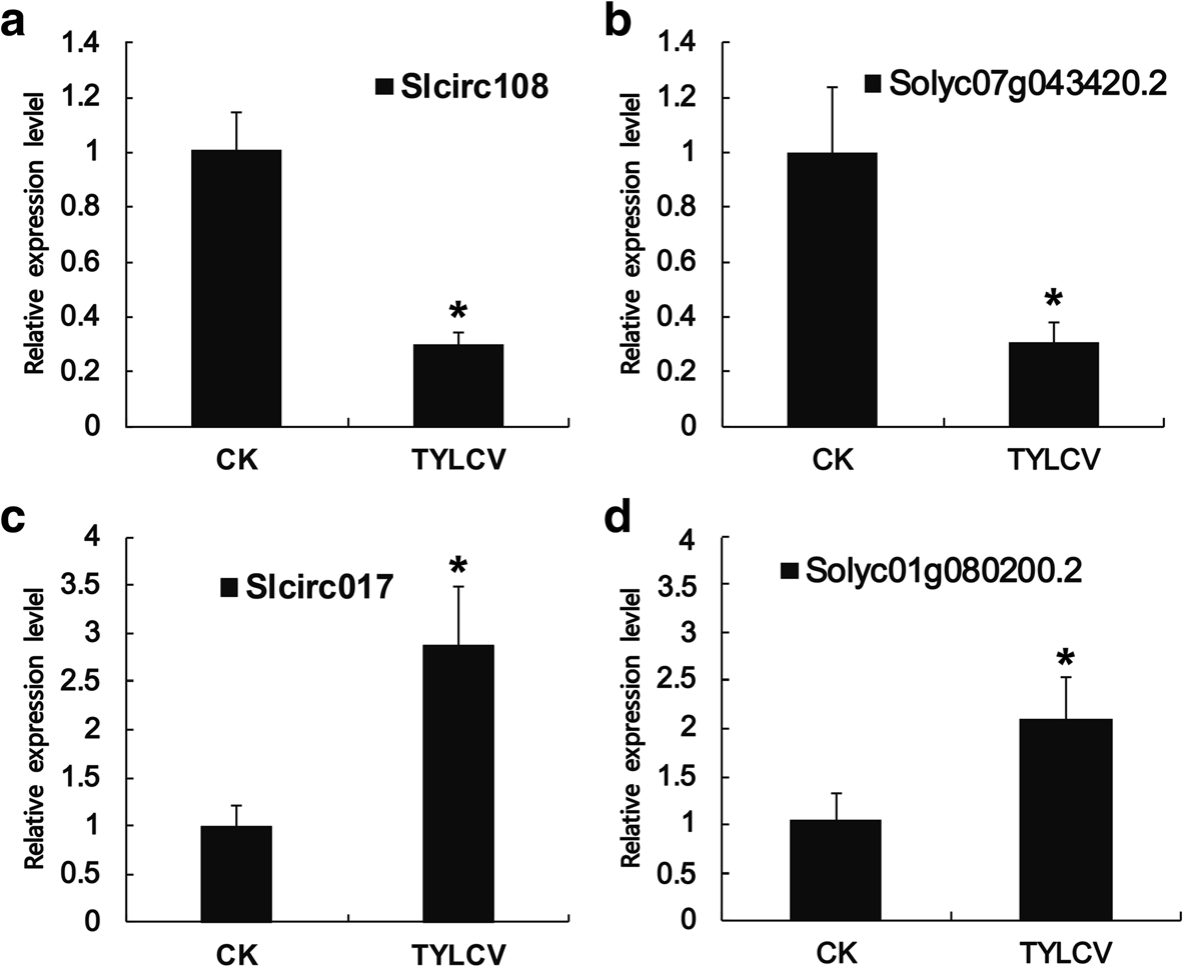
The expression pattern between circRNAs and parent genes. **a** Relative expression level of Slcirc108. **b** Relative expression level of Slcirc108 parent gene Solyc07g043420.2. **c** Relative expression level of Slcirc017. **d** Relative expression level of Slcirc017 parent gene Solyc01g080200.2

In a previous study, exon-intron circRNAs were found to up-regulate the expression of their parent protein-coding genes [49]. We investigated whether exonic circRNAs have disparate effects on expression correlations between exonic circRNAs and their parent genes in CK and TYLCV. The expression of Slcirc108 was down-regulated significantly after TYLCV infection (Fig. 9a), and the expression of parent gene Soly07g043420.2 was also down-regulated (Fig. 9b). In addition, expressions of Slcirc017 and parent gene Solyc01g080200.2 were up-regulated after TYLCV infection. These results suggested that the expression of exonic circRNAs were correlated with the expressions of parent genes.

### Gene silencing validation of circRNA parent genes following TYLCV infection

**Fig. 10:**
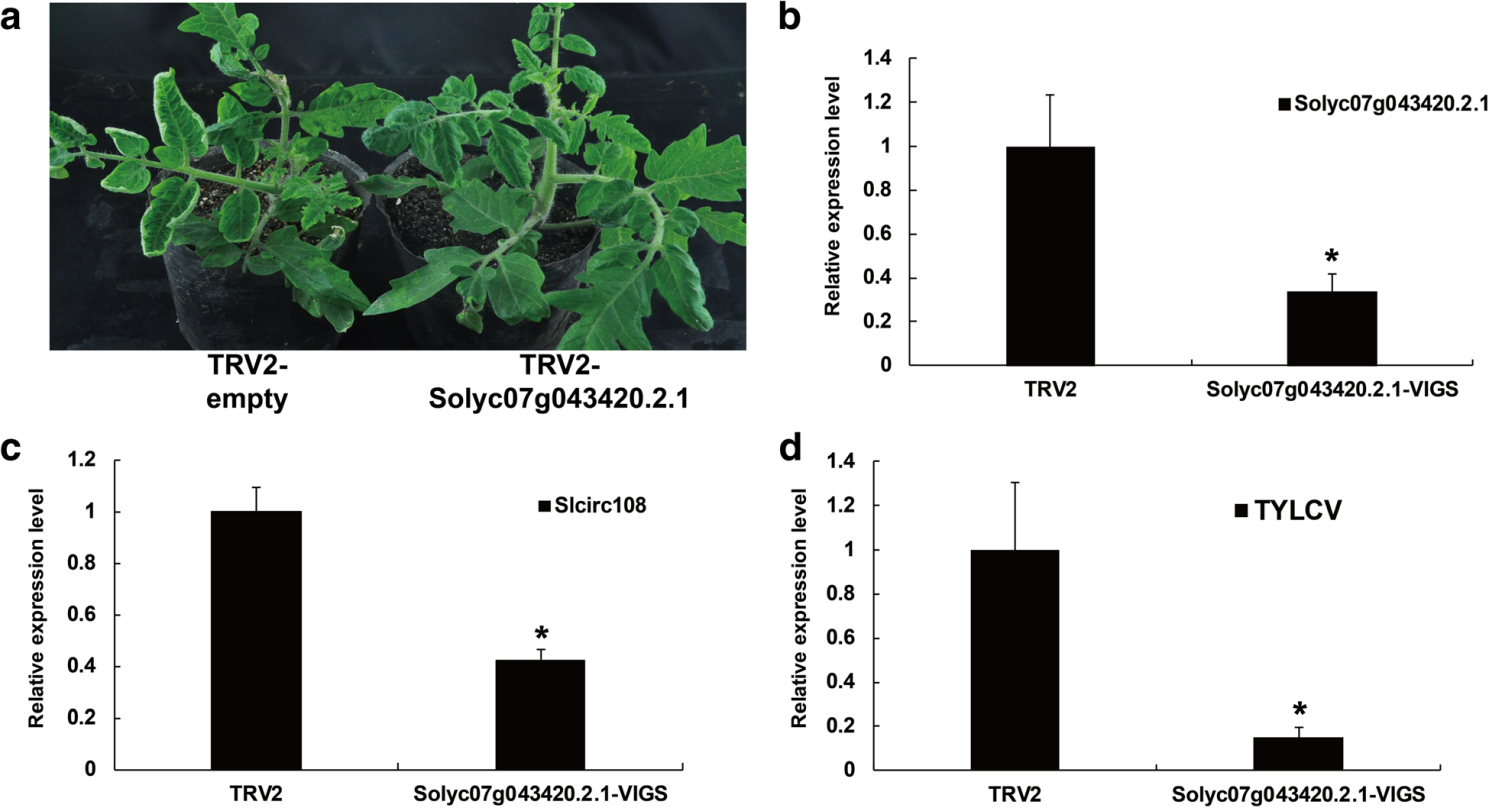
Validation of Slcirc108 parent gene Solyc07g043420.2.1 with virus-induced gene silencing. **a** Cotyledon agroinfiltration of TRV vectors with TYLCV infection was performed in the susceptible tomato at the cotyledon stage. Plants treated with the pTRV empty vectors showed the susceptible phenotype (Left). Susceptible plantlets treated with the pTRV1 and pTRV2-Solyc07g043420.2.1 had no symptoms (right). **b** Relative expression levels of Solyc07g043420.2.1 using real-time RT-PCR analysis in the VIGS-treated tomato plants 20 days after agroinfiltration with TRV2 vectors. **c** Relative expression levels of Slcirc108 using real-time RT-PCR analysis in the VIGS-treated tomato plants 20 days after agroinfiltration with TRV2 vectors. **d** TYLCV accumulation in the Solyc07g043420.2.1 silenced plants was estimated from the total genomic DNA by quantitative RT-PCR

To investigate the effect to TYLCV, the circRNA Slcirc107 parent gene Solyc07g043420.2.1 was further challenged with TYLCV after VIGS at the cotyledon stage for functional characterization. One month after agroinfiltration, tomato plants injected with TRV-Solyc07g043420.2.1 showed partial resistant to TYLCV compared to TRV control plants (Fig. 10a). For TRV control, the expression of Solyc07g043420.2.1 and circRNA Slcirc108 decreased by 40% (Fig. 10b and c). Meanwhile, total genomic DNA from TYLCV-infected tomato plants was extracted for the detection of virus accumulation. Quantitative RT-PCR revealed that the TYLCV accumulation decreased five-folds in VIGS-treated tomato plants compared with the negative control (Fig. 10c). These results suggested that circRNA parent genes were involved in the response to TYLCV infection.

## Discussion

LncRNAs have emerged as potential key regulators of gene expression, due to their involvement in many developmental, biotic and abiotic processes. A great deal of lncRNAs have recently been identified in tomatoes and act as competing Endogenous target mimics (eTMs) for tomato microRNAs involved in TYLCV infection [13]. Meanwhile, thousands of tomato lncRNAs were identified in fruit ripening, and the silencing of two novel lncRNAs resulted in delay of ripening [14]. In this study, a total of 2056 lncRNAs were identified in susceptible tomato lines infected with TYLCV. Although the criteria for identification of tomato lncRNAs was similar to the previous study, a higher number of lncRNAs as well as specifically expressed lncRNAs were identified in susceptible tomatoes. There might be three main reasons for this: (i) a ribosome-depleted library construction was used in this study, which suggested that lncRNAs without poly(A) tails were obtained; (ii) strand-specific RNA-seq allowed identification for transcription orientation of some lncRNAs; (iii) the larger data set obtained allowed detection of more lncRNAs with low abundance.

eTMs with miRNAs is a regulatory mechanism of lncRNA. It has been shown that lncRNAs act as miRNA sponge that contain miRNA-binding sites which have been indicated to regulate miRNAs and their targets [50]. In Arabidopsis, 36 lncRNAs were identified as endogenous target mimic for 11 conserved miRNAs, and eTMs of miR160 and miR166 are functional in the regulation of plant development [50]. In rice, several lncRNAs were also identified as competing endogenous RNAs, which bound miR160 and miR164 in a type of target mimicry [11]. In a previous study, tomato lncRNAs slylnc0195 and slylnc1077 acted as competing eTMs for tomato miR166 and miR399, respectively [13]. In our study, S-slylnc1484 was predicted to be a ‘decoy’ for tomato miR399, while no lncRNAs were predicted as eTM of miR166. These results indicated that the miR399-lncRNA pair might be contributing to an important regulatory pattern in both susceptible and resistant tomatoes following TYLCV infection, and the miR166-lncRNA pair is just involved in the resistance to TYLCV infection.

Many lncRNAs have been proved to play important roles in plant-pathogen interaction. In wheat, four lncRNAs were identified from bread wheat lines infected with *Puccinia striiformis* f. sp. Tritici (*Pst*). Expression analysis revealed that lncRNAs play a crucial role in the resistance mechanism of plants, indicating mechanisms that regulate defense pathways to stripe rust [51]. In addition, 159 novel intergenic lncRNAs, including 20 *Fusarium oxysporum* infection responsive lncRNAs were identified, and some of them are direct targets of transcription factors responsive to pathogen attack [52]. In tomatoes, 688 differentially expressed lncRNAs were identified between *Phytophthora infestans*-resistant and -susceptible tomato lines, and among them lncRNA16397 acted as an antisense transcript of SlGRX22 to regulate its expression, which can reduce reactive oxygen species accumulation and alleviate cell membrane injury, thus enhancing tomato resistance to *P. infestans* [15]. In our study, a total of 2056 lncRNAs (1767 lincRNAs and 289 lncNATs) were identified in the susceptible tomato line following TYLCV infection, and silencing of differentially and up-regulated expressed lncRNAs can enhance resistance to TYLCV by VIGS. These findings could shed new light on lncRNAs by silencing their expression in susceptible tomato plants to increase TYLCV resistance, except for traditional introgression of resistant genes into susceptible plants.

Recently, circular RNAs have been discovered in types of mammal cells, and often show tissue/developmental-stage-specific expression. In plants, circRNAs have been identified in Arabidopsis and rice by genome-wide analysis [27, 37]. In tomatoes, 854 circRNAs were predicted and 163 of them exhibited chilling responsive expressions, but their features were not clear [40]. In this study, we conducted a genome-wide identification of circRNA in tomato plants following TYLCV infection. Our results indicated the presence of circRNAs in tomatoes by using CIRI, and validation by PCR and Sanger sequencing. Compared with other plant species, such as Arabidopsis and rice, the total number of circRNAs in tomatoes were relatively lower, which might be attributed to the more restrictive filter criteria in our study.We manually filtered 1151 circRNAs observed with lower than 10 reads. In rice and Arabidopsis, exonic circRNAs exhibited the highest proportion (50.5 and 85.7%) [27], and this trend was consistent in tomato plants (62%). Additionally, expression patterns in CK and TYLCV samples were different, thereby indicating the diverse roles of tomato circRNAs in different biological processes. In our study, we also used additional software to predict circRNAs, such as circRNA_finder, CIRIexplorer, and MapSplice. However, we found that these software programs could not predict a higher number of circRNAs than the CIRI program. These results suggested that CIRI might be more suitable for circRNA prediction in plants.

In rice, the expression of exonic circRNAs was significantly and positively correlated to their parent genes [27]. This correlation pattern was also observed in kiwifruit [39]. In this study, the expression of circRNAs positively correlated with their parent genes (Fig. 9), indicating that most circRNAs can up- and down-regulate the expression of their parent genes, and act as an “enhancer” of their parent genes. However, the regulatory mechanism of this expression pattern need to be further investigated.

It has been reported that circRNAs play important roles in regulating gene expression by acting as a miRNA sponge and preventing them from degrading the targets mRNAs [38, 40, 41]. To identify whether tomato circRNAs could affect post-transcriptional regulation of mRNA by binding to miRNAs in our study, psRobot algorithms were used to identify circRNA-derived target mimics [53]. However, no circRNAs were bound to tomato miRNAs following bioinformatics prediction. To further validate the function of circRNA with TYLCV infection, we silenced the Slcirc107 parent gene Solyc07g043420.2.1 using VIGS method. We found that the accumulation of TYLCV was significantly decreased compared with the TRV control, and the VIGS plants showed resistance to TYLCV. These results suggested that circRNAs and their parent gene were both involved in TYLCV infection.

## Conclusion

Previous studies have illustrated that lncRNAs play important roles in TYLCV infection in resistant tomato plants. The results of the present study indicate that some novel lncRNAs are differentially inducible as negative regulators, and gene silencing of lncRNAs can promote tomato resistance to TYLCV infection. Moreover, we identified 184 circRNAs by CIRI software, and found that the expressions of exonic circRNAs were correlated with the expressions of parent genes. In summary, genome-wide identification of long non-coding RNA and circRNAs in susceptible tomato plants and analysis of their features and functions with TYLCV infection suggested that selected lncRNAs and circRNAs are a critical class of noncoding regulators in tomato. Further functional characterization of lncRNAs and circRNAs in TYLCV infection are warranted to elucidate the underlying mechanisms for these RNAs.

## Methods

### Plant growth, viral inoculation and detection

The TYLCV-susceptible tomato breeding line JS-CT-9210 was grown in insect-proof environment under 25 °C and 16 h light/8 h dark. The TYLCV infectious clone was provided by Xueping Zhou (Zhejiang University, China) [54]. Tomato plants at the two to three-leaf stage were injected with the TYLCV infectious clone. To confirm the success of TYLCV infection in the susceptible tomato plants, DNA was extracted from young leaves of infected and untreated tomato plants at 21 days post inoculation (dpi), and TYLCV accumulation was detected by quantitative RT-PCR. The detailed procedures were referenced in a previous study [13]. The primers used for this study are listed in Additional file 8: Table S4.

### Plant sampling and sample sequencing

Leaf samples infected with TYLCV clones were collected at 7 days post inoculation (dpi) and flash frozen immediately in liquid nitrogen. At 21 dpi, the tomato plants with samples collected were observed to have virus symptoms. Samples with obvious virus symptoms and high TYLCV titer were selected to perform RNA-seq. Three independent biological replicates each of TYLCV-infected and untreated JS-CT-9210 tomato leaves were used for RNA sequencing. Due to some lncRNAs lacking polyA tails, the rRNA of total RNA was removed using the Ribo-Zero rRNA Removal Kits (Plant) according to the manufacturer’s instructions (Illumina, USA). Total RNA was treated to remove rRNA and the quality of the RNA was detected using the Agilent 2100 Bioanalyzer. Six strand-specific RNA libraries were prepared using a UTP method [55], and submitted to Annoroad Corporation (Beijing, China) for 150 bp pair-end sequencing on the Hiseq 4000 (Illumina, USA) at a depth of 120 million reads (Table 1).

### Bioinformatic identification of lncRNA

LncRNAs were identified according to previous study, and some steps were modified in this study [13]. Primarily, antisense transcripts or intergenic transcripts with fragments per kilobase of transcript per million mapped reads (FPKM) that were recorded as higher than 1 in a single exon or 2 in multiple exons in at least one sample were scored as lncRNA candidates. Additionally, CPC and CNCI algorithms were used to calculate the coding potential of transcripts. Transcripts without coding potential were aligned with non-redundant (NR) protein database to exclude transcripts with significant homology to known coding proteins. We also compared the lncRNAs in this study with those identified in resistant tomato plants in the previous study [13]. All lncRNAs sequences in the resistant tomato plants were downloaded (https://www.nature.com/articles/srep16946) for further analyzation.

### Computational identification of circular RNA

The clean reads of three biological replicates in CK and TYLCV samples were assembled into two data libraries, respectively. The two libraries were aligned to the tomato genome assembly build 2.50 using BWA-MEM software [56]. The circRNAs were predicted and identified by the CIRI algorithm with genomic annotations from International Tomato Annotation Group 2.4 (ITAG2.4) according to instruction [34]. The circRNA internal structure was identified using the CIRI-AS program [57].

### Prediction of the function of lncRNAs

Previous studies have reported that lncRNAs can regulate the expression of neighboring protein coding genes. Thus, the localization of lncRNAs and coding genes were analyzed to identify a co-located pair separated by less than 100 kb [58]. Gene Ontology (GO) of coding genes and differentially expressed genes (DEGs) were analyzed using Blast2GO. Furthermore, pathway enrichment analysis of these genes was performed using the Kyoto Encyclopedia of Genes and Genomes (KEGG) database [59].

### Differential expressions of lncRNAs and circRNAs between CK and TYLCV samples

The change in lncRNA expression was calculated as the fold change (FC) in FPKM between CK and TYLCV samples. Only the lncRNAs that met the criteria of log2 FC ≥ 1 or ≤ − 1 with *P* value < 0.05 were considered differentially expressed. In addition, raw BSJs reads for two samples were normalized to the total reads number and log2 subsequently transformed as differentially expressed circRNAs using a fold change cutoff of 1 between CK and TYLCV samples.

### Validation of differentially expressed lncRNAs and circRNAs

qRT-PCR was used to validate the expressions of lncRNAs and circRNAs identified by earlier bioinformatic analysis. Eight differentially expressed lncRNAs and two exon-derived cirRNAs were selected for experimental validation. Two micrograms of total RNA were treated with Dnase I before revere transcription PCR. Primers used for the quantitative RT-PCR analysis of lncRNAs were designed using the Beacon Designer 7.5 software (Premier Biosoft International, Palo Alto, California, USA). The primers for circRNAs were designed using the “Out-facing” strategy to guarantee amplification [60]. And the primers of the exonic circRNA parent genes were also designed by the Beacon Designer 7.5 software. Primers sequences are listed in Additional file 8: Table S4.

### Validating circRNAs by PCR and sanger sequencing

To validate the identified circRNAs in tomatoes, we extracted total RNA and genomic DNA of tomato leaves. The first-strand cDNA was synthesized from 1μg of total RNA with random primers using the HiScript II One Step RT-PCR (Vazyme, Nanjing, China). Seven circRNAs were randomly selected for validation of PCR and Sanger sequencing. The divergent and convergent primers were designed according to a previous study [37]. The primers sequences are listed in Additional file 8: Table S4.

### Virus-induced gene silencing (VIGS) of tomato lncRNAs and exon-circRNA parent gene and TYLCV accumulation in VIGS-tomato

Tobacco rattle virus (TRV) mediated VIGS was used to silence lncRNAs and exon-derived circRNAs. TRV is a bipartite, positive-strand RNA virus with the TRV1 and TRV2 genomes. To induce post-transcriptional gene silencing (PTGS), the TRV2 genome is genetically modified to carry a fragment of the target gene and delivered into the plant (along with the TRV1 genome) by agroinfiltration. [13]. Briefly, pTRV-containing Agrobacterium EHA105 was cultured in liquid LB medium with kanamycin and rifampin overnight at 28 °C. Agrobacterium cells were harvested and resuspended in infiltration media (10 mM MgCl_2_, 10 mM MES, 200 mM acetosyringone) to an O.D.value of 2.0 and cultured at room temperature for 4 h. For agroinfiltration, an equal volume of Agrobacteria containing of pTRV1 and pTRV2- lncRNAs or exon-derived circRNAs was mixed and infiltrated into the cotyledons of tomato seedlings at the cotyledon stage with 1 ml syringe. The agroinfiltration of pTRV1 with pTRV2-PDS and pTRV1 with empty pTRV2 served as positive control and negative control, respectively. As a positive control, the phytoene desaturase (PDS) gene can be targeted for VIGS. PDS-silenced plants show visible bleached leaves due to disruption of phytoene desaturation, which is an important step in the β-carotene biosynthesis pathway. The primer sequences in this part are listed in Additional file 8: Table S4.

## Additional files

Additional file 1: **Figure S1.** Quality for each base in reads viewed by software FastQC. (JPG 4943 kb)

Additional file 2: **Figure S2.** Comparison of gene expression in any two libraries of three repeats. The Pearson’s correlation (R value) was calculated between the log2-transformed FPKM values of two libraries. (JPG 12188 kb)

Additional file 3: **Table S1.** Characteristics of all lincRNAs identified in this study. (XLSX 181 kb)

Additional file 4: **Table S2.** Characteristics of all lncNATs identified in this study. (XLSX 39 kb)

Additional file 5: **Figure S3.** The statistics of Pathway enrichment of flanking gene of long non-coding RNA. (JPG 3122 kb)

Additional file 6: **Figure S4.** Expression levels determined by RNA-Seq and qRT-PCR are highly correlated. (JPG 231 kb)

Additional file 7: **Table S3.** Characteristics of all circular RNAs identified in this study. (XLSX 27 kb)

Additional file 8: **Table S4.** Primers list in this study. (XLSX 16 kb)

Additional file 9: **Figure S5.** The structure and Sanger sequencing of circular RNA in tomato. (JPG 9849 kb)

## Abbreviations

AS: Alternative splicing
bHLH: Basic helix-loop-helix
BSJs: Back-spliced junctions
circRNAs: Circular RNAs
CIRI: CircRNA Identifier
CNCI: Coding-Non-Coding Index
COLDAIR: Cold-assisted intronic non-coding RNA
COOLAIR: Cool-assisted intronic non-coding RNA
CPC: Coding Potential Calculator
DEGs: Differentially expressed genes
DELs: Differentially expressed lncRNAs
dpi: Days post inoculation
ER: Endoplasmic reticulum
eTMs: Endogenous target mimics
FC: Fold change
FLC: Flowering Locus C
FPKM: Fragments per kilobase of transcript per million mapped reads
GO: Gene Ontology
HID1: Hidden treasure 1
ITAG2.4: International Tomato Annotation Group 2.4
KEGG: Kyoto Encyclopedia of Genes and Genomes
lincRNAs: Long intergenic non-coding RNA
lncNATs: Long non-coding natural antisense transcripts
LncRNAs: Long Noncoding-RNAs
miRNA: microRNA
ncRNAs: Non-coding RNAs
NR: Non-redundant
Pol II: RNA polymerase II
RDRs: RNA-dependent RNA polymerases
siRNA: Small interfering RNA
tasiRNA: *trans*-acting siRNA
TRV: Tobacco rattle virus
TYLCV: Tomato yellow leaf curl virus
VIGS: Virus-induced gene silencing
