# Supplementary figures and images for: Re-analysis of long non-coding RNAs and prediction of circRNAs reveal their novel roles in susceptible tomato following TYLCV infection
Source: BMC Plant Biol. 2018 Jun 4;18:104. doi: 10.1186/s12870-018-1332-3 (PMC5987537; doi:10.1186/s12870-018-1332-3)

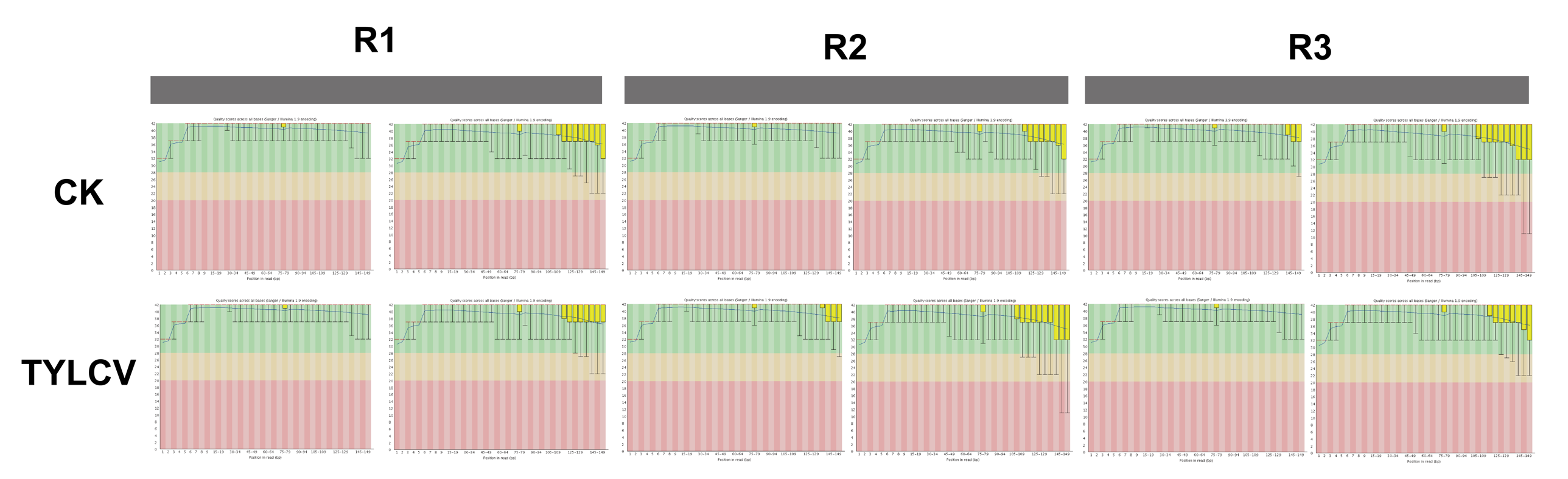

Supplement: Supplementary file 1 — Figure S1. Quality for each base in reads viewed by software FastQC. (JPG 4943 kb) [file 12870_2018_1332_MOESM1_ESM.jpg]

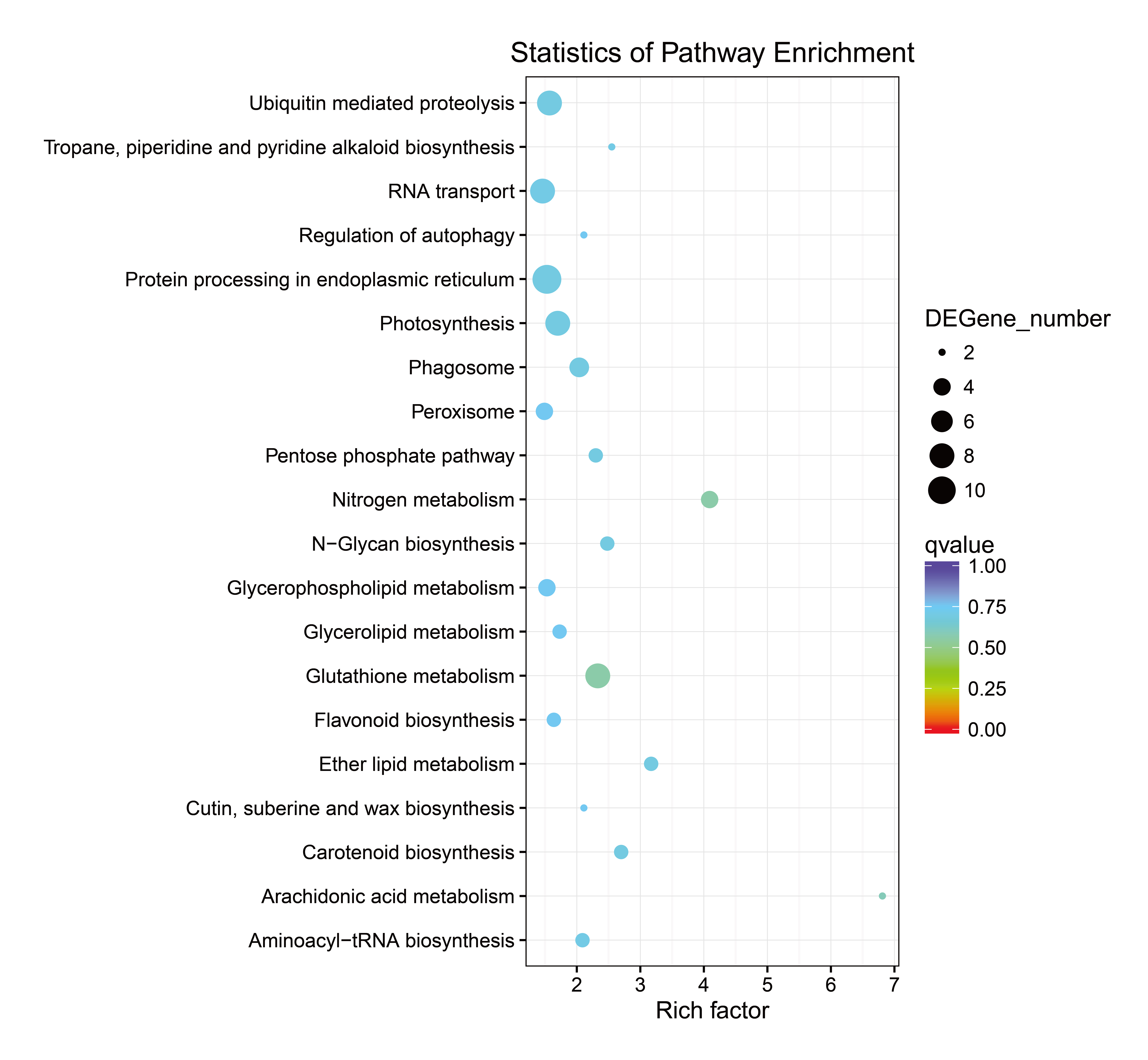

Supplement: Supplementary file 5 — Figure S3. The statistics of Pathway enrichment of flanking gene of long non-coding RNA. (JPG 3122 kb) [file 12870_2018_1332_MOESM5_ESM.jpg]

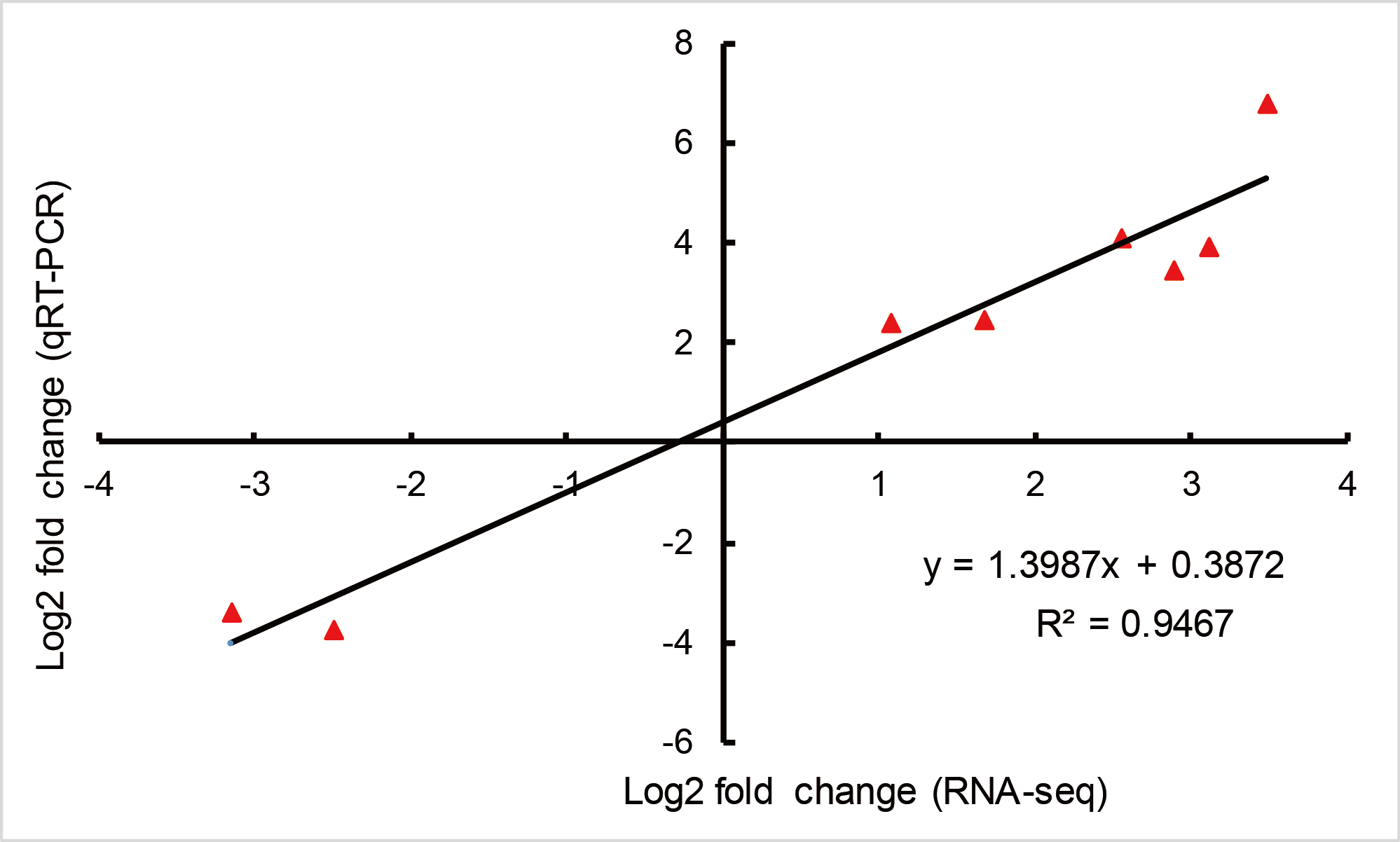

Supplement: Supplementary file 6 — Figure S4. Expression levels determined by RNA-Seq and qRT-PCR are highly correlated. (JPG 231 kb) [file 12870_2018_1332_MOESM6_ESM.jpg]
